# Trade-Off between Degradation Efficiency and Recyclability: Zeolite-Enhanced Ni_3−x_Co_x_S_4_ Catalyst for Photocatalytic Degradation of Methylene Blue

**DOI:** 10.3390/molecules29174167

**Published:** 2024-09-03

**Authors:** Rachel Anne E. Lagunay, Ritche Roi B. Adalim, Aruzhan Tleubekova, Diana Suleimenova, Marvin Jose F. Fernandez, Robert J. O’Reilly, Mannix P. Balanay

**Affiliations:** 1 Department of Chemistry, Nazarbayev University, Astana 010000, Kazakhstan; rachelanne.lagunay@nu.edu.kz; 2 Department of Chemistry, Mindanao State University-Iligan Institute of Technology, Iligan City 9200, Philippines; ritcheroi.adalim@g.msuiit.edu.ph (R.R.B.A.); marvinjose.fernandez@g.msuiit.edu.ph (M.J.F.F.); 3 Department of Biology, Nazarbayev University, Astana 010000, Kazakhstan; aruzhan.tleubekova@nu.edu.kz; 4 National Laboratory Astana, Nazarbayev University, 53 Kabanbay Batyr Ave., Astana 010000, Kazakhstan; www.lady.di@mail.ru; 5 Department of Chemistry, L.N. Gumilyov Eurasian National University, 2 Satpayev St., Astana 010008, Kazakhstan; 6 School of Science and Technology, University of New England, Armidale, NSW 2351, Australia

**Keywords:** nickel cobalt sulfides, zeolite, UV, artificial sunlight, pseudo-first order, pseudo-second order

## Abstract

We herein report successful syntheses of both nickel cobalt sulfide (NCS) and its composite with zeolite (NCS@Z) using a solvothermal method. Techniques such as EDX analysis, SEM, and molar ratio determination were used for product characterization. The incorporation of NCS significantly changed the surface roughness and active sites of the zeolite, improving the efficiency of methylene blue degradation and its reusability, especially under UV irradiation. In comparing the pseudo-first order rates, the highest degradation efficiency of methylene blue was achieved with NCS-2@Z, having a degradation extent of 91.07% under UV irradiation. This environmentally friendly approach offers a promising solution for the remediation of methylene blue contamination in various industries.

## 1. Introduction

Dyes are compounds that are known for their bright colors; this property arises because of the existence of a chromophore, allowing such compounds to absorb light in the visible spectrum, typically between 400 and 700 nanometers. When incorporated into materials, these compounds provide a wide range of long-lasting colors that tend to withstand exposure to water, light, oxidizing agents, perspiration, and microbial interactions [1]. This versatility has led to their use in various settings, including the textile industry [2,3,4], the leather industry [5], hair dyeing [6], and even the food industry [7]. In addition, dyes are also commonly employed in agricultural research [8], light harvesting arrays [9], and photoelectrochemical cells [10], and also play a central role in fields such as medicine, electronics, and the non-impact printing industry [11]. However, the widespread production of synthetic dyes, estimated at around 10,000 tons per year worldwide, is fraught with environmental concerns [12]. One notable problem is their longevity in the environment due to their recalcitrant nature, which leads to difficulties in biodegradation and removal. As these dyes are water-soluble, they pose a contamination risk if they enter water bodies in an untreated form, which can adversely affect aquatic life, animals, and humans [12,13].

**Figure 1:**
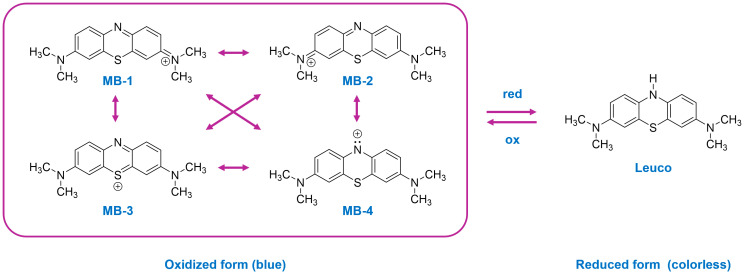
Comparison of resonance structures depicting the oxidized form of MB alongside its reduced leuco form.

One of the most widely used synthetic dyes is 3,7-bis(dimethylamino)-pheno-thiazine chloride tetramethylthionine chloride, commonly known as methylene blue (MB), a water-soluble cationic aromatic heterocyclic-based dye with a pK_a_ of ~3.8 [14,15]. The structure of cationic MB is a combination of several valence bond resonance forms, as shown in Figure 1 [16]. Upon reduction, MB adopts a colorless leuco form (Figure 1), which has a higher pK_a_ (5.8), as well as a particularly low ionization potential at physiological pH. The redox properties of MB result in it possessing desirable pharmacological properties. For example, in addition to being investigated for use in the treatment of Alzheimer’s disease, it is also employed as an antimalarial agent, as well as an antimicrobial, chemotherapy, and blood disinfection agent [17,18]. In contrast to the use of MB in medicine, where it is administered at particularly low concentrations, the concentrations used in industry, where it is used to dye materials such as textiles and cosmetics, are significantly greater. Consequently, due to industrial activity, industrial effluent containing large quantities of MB may be discharged into water bodies (such as the ocean, rivers, or lakes), which poses a significant environmental challenge. Untreated or poorly treated effluent containing higher concentrations of MB can cause a number of problems in humans, including, for example, cyanosis, tissue necrosis, the formation of Heinz bodies, vomiting, jaundice, dyspnea, shock, methemoglobinemia, and tachycardia [14].

The urgency of addressing these environmental challenges requires the rapid implementation of efficient remediation methods using economically viable approaches and materials. Conventional methods such as flocculation, sedimentation, and adsorption have proven to be ineffective due to the solubility and stability of dyes, such as MB, and often only lead to a transfer of compounds between phases without significant degradation of the dye itself. In response to this, photocatalysis is proving to be a promising solution that enables sustainable and effective degradation of organic compounds upon exposure to visible or ultraviolet (UV) light. Nanoparticles (NPs) play a central role in this process and are divided into three generations [19]. The first generation includes NPs made of metals or metal oxides such as Ag, Au, Pd, TiO_2_, and ZnO, while the second generation includes binary and ternary metal oxides [20,21]. The third generation, however, is the most promising as it contains a mixture of NPs or combines them with different materials such as polymers, carbon-based materials, metal–organic frameworks, and naturally occurring zeolites [22]. These customized combinations provide a significant surface area, increasing the catalytic activity for efficient dye degradation. Among the composites, zeolites have proven to be particularly advantageous due to their inherent physical and chemical properties. In this regard, the fact that zeolites consist of an aluminosilicate crystal framework renders them particularly useful in wastewater treatment, while the fact that they are also relatively cheap materials means that their use in water treatment is economically advantageous. The modification of zeolite structures can further improve their adsorption capacity, making them very efficient in wastewater treatment [23,24]. Furthermore, the incorporation of semiconductor materials into zeolite frameworks can further enhance their photocatalytic capabilities. For example, Bagale and colleagues [25] found that a zeolite-supported CdS/TiO_2_/CeO_2_ composite material was able to degrade MB with an efficiency of 99.9%, whereas a bare ternary photocatalyst achieved <80% degradation. In another study, a zeolite/TiO_2_/Ni nanocatalyst was able to degrade dyes with an efficiency of 99.8% [26]. Ternary photocatalysts have great potential for the degradation of dyes, but also have some drawbacks, including the need for complicated synthetic procedures, optimization problems, and interfacial compatibility issues. These shortcomings contribute to the high cost of their application. However, overcoming these hurdles is crucial to advancing sustainable solutions in photocatalysis. One promising approach is to explore binary transition metal complexes, which are abundant in the Earth’s crust, as alternatives. These materials can be obtained via simpler synthetic routes and may offer improved compatibility, as well as lower production costs, all the while maintaining their effectiveness in environmental remediation. Nickel cobalt sulfide (NCS) and its composites with zeolites are proving to be particularly promising photocatalysts for the degradation of dyes. Research has shown that composites based on NCS exhibit enhanced photocatalytic activity due to their superior electrical conductivity and unique crystal structures [27,28]. NCS@MXene composites in particular have shown exceptional stability and efficacy in degrading dyes under visible light [29]. In addition, co-catalysts such as NiCo_2_S_4_/CdS [30], NiCo_2_S_4_@Zn_0_._5_Cd_0_._5_S [31], and NiCo_2_S_4_/g-C_3_N_4_ [32] have been shown to be effective in the generation of hydrogen by photocatalysis under visible light, highlighting the broad potential of NCS-based materials in various photocatalytic applications. Moreover, NiCo_2_S_4_ micro-particles were synthesized via a solvothermal method and then applied as noble-metal-free catalysts in a CO_2_ photoreduction system [33]. In this context, the synthesis of NCS and its composites with zeolite using solvothermal methods was proven to be a promising route for efficient dye degradation. This present study aimed to explore the application potential of NCS-based photocatalysts, focusing on the degradation of MB, and provides insights into sustainable strategies to mitigate synthetic dye wastes.

## 2. Results and Discussions

The catalytic performance of Ni/Co/S can be affected by various factors, including the specific synthesis method for different Ni/Co/S compositions. Researchers have found that altering these compositions leads to variations in specific capacitance values [34,35,36,37]. This observation led us to examine how these compositional differences influence the material’s catalytic performance. In this study, we investigated Ni/Co ratios of 2:1 and 1:2 to explore their effects, herein labeled as NCS-1 and NCS-2, respectively.

### 2.1. Characterization of the NCS-1 and NCS-2 Catalysts

**Figure 2:**
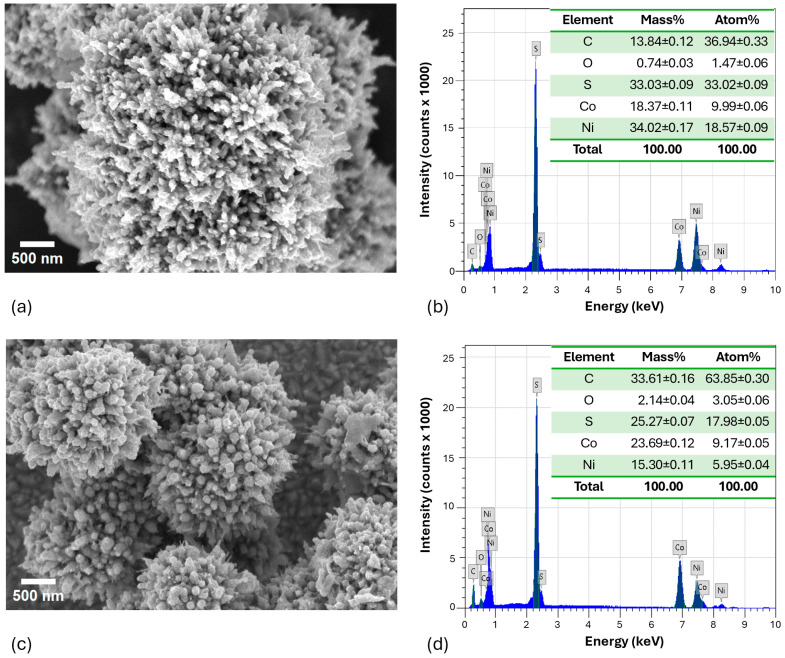
SEM images and EDX spectra with the elemental compositions of (**a**,**b**) NCS-1 and (**c**,**d**) NCS-2.

The morphological surface properties of nickel cobalt sulfide (NCS) were investigated using scanning electron microscopy, as illustrated in Figure 2a,c. Corresponding elemental analyses were conducted through energy dispersive X-ray spectroscopy, with the results shown in Figure 2b,d. Both NCS-1 and NCS-2 exhibited tiny spherical shapes that clustered together to form larger spherical structures. Notably, NCS-2 formed smaller clusters compared to NCS-1. This difference resulted in a larger surface area for NCS-2, which typically enhances photocatalytic activity by providing more active sites for reactions. Additionally, smaller clusters may improve light scattering and absorption efficiency, potentially leading to superior photocatalytic performance. The EDX spectra confirmed that the synthesized materials contained the intended elements, with any carbon detected attributable to the conductive carbon adhesive tape used during sample mounting.

**Figure 3:**
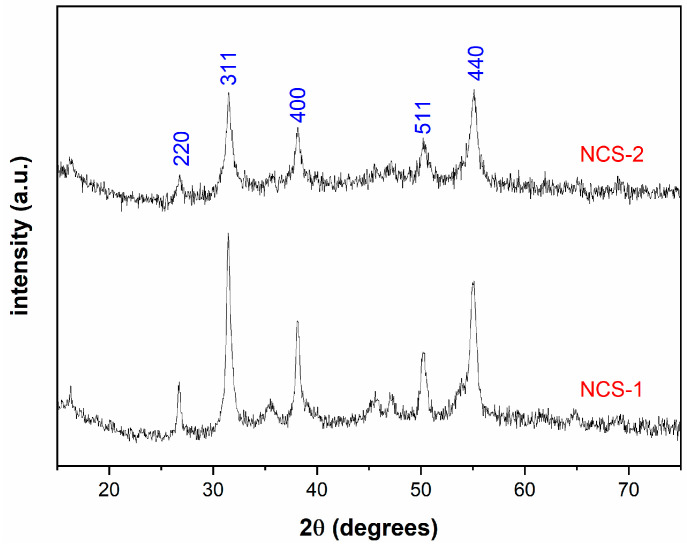
XRD spectra of NCS-1 and NCS-2.

The crystallographic structures of NCS-1 and NCS-2 were evaluated by examining the diffraction peaks in the XRD spectra, as depicted in Figure 3. This figure showcases distinct peaks observed in the synthesized samples at 2θ values of 26.7°, 31.5°, 38.2°, 50.2°, and 55.2°, corresponding to the crystalline planes (220), (311), (400), (511), and (440), respectively, from a cubic NiCo_2_S_4_ (JCPDS card no. 020-0782) [38]. Remarkably, both 2:1 and 1:2 Ni/Co molar ratios yielded the same product with no noticeable shifts, consistent with the observations from other studies [34,36].

### 2.2. Dye Degradation Investigations on NCS-1 and NCS-2 Catalysts

#### 2.2.1. Effect of Light Conditions

In the investigation of the degradation of MB by H_2_O_2_ alone, the initial tests showed minimal degradation when 1 mL of 30% H_2_O_2_ was applied. The effect of the different light conditions on the degradation process was then investigated using the synthesized materials. This study included the effects of UV light (254 nm), light, and darkness, each at different contact times from 2 to 15 min and a catalyst dosage of 15 mg. Assuming pseudo-first order kinetics for MB degradation, the rate constant, *k*_1_, was calculated using the natural logarithmic plot of (*A*/*A_o_*) versus time, as shown in Equation (2).

(1) $$\ln\left(\frac{A}{A_{o}}\right)=-k_{1}t$$

**Figure 4:**
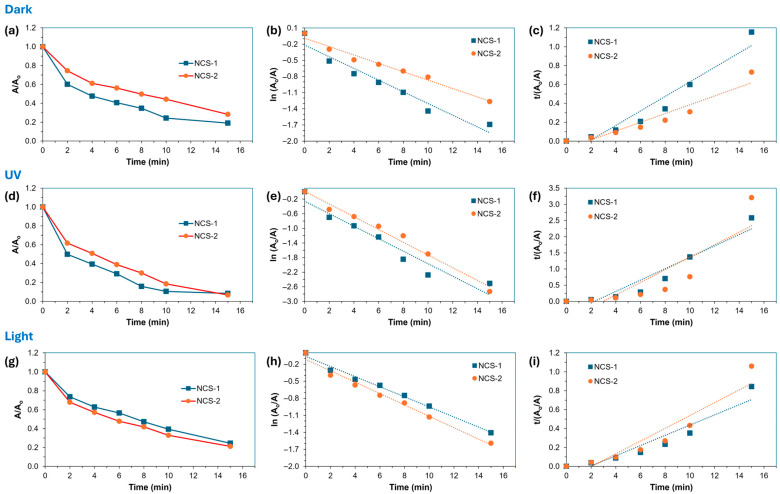
(**a**,**d**,**g**) Influence of initial MB concentration on the photocatalytic degradation efficiency of NCS-1 and NCS-2 catalysts. Plots illustrating the (**b**,**e**,**h**) pseudo-first order and (**c**,**f**,**i**) pseudo-second order kinetics for the degradation of MB utilizing NCS-1 and NCS-2 catalysts under different light conditions. The dotted lines in the kinetic plots represent linear regression fittings of the experimental data.

In addition, a pseudo-second order kinetic model was tested using Equation (3),

(2) $$\frac{t}{A_{t}}=\frac{1}{k_{2}A_{e}^{2}}+\frac{t}{A_{e}}$$

where *A_e_* represents the equilibrium extinction of the dye. The rate constant, *k*_2_, was derived from the square of the slope divided by the intercept of the *t*/*A_t_* versus *t* plot. The resulting data are summarized in Table 1 and the corresponding plots are shown in Figure 4. To determine the best fit to the experimental data, the linear regression coefficients (*r*^2^) were compared for all catalysts under the different light conditions. It is noteworthy that the rate constants obtained with the pseudo-first order model exceeded those of the pseudo-second order model, indicating a faster reaction rate under the conditions assumed by the former. The pronounced pseudo-first order linearity became evident when the mixture was exposed to either UV radiation or light, indicating specific wavelengths or energy levels within these light sources that favored this behavior. This observation is generally due to the addition of H_2_O_2_ in the system, which creates a photo-Fenton-like environment. In this system, H_2_O_2_ decomposes in the presence of a catalyst and generates hydroxyl (•OH) radicals.

#### 2.2.2. Effect of Amount of Catalyst

**Figure 5:**
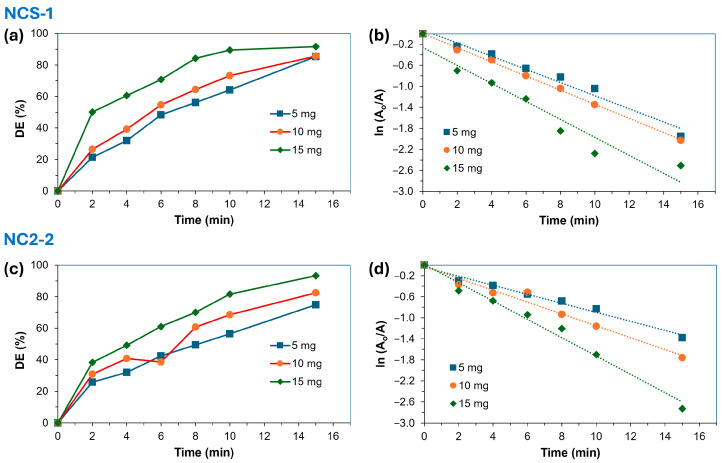
(**a**,**c**) Influence of the amount of NCS-1 and NCS-2 catalysts on the degradation efficiency (%) of MB and the diagrams of the (**b**,**d**) pseudo-first order degradation of MB using different amounts of NCS-1 and NCS-2 catalysts under UV irradiation (254 nm). The dotted lines in the kinetic diagrams represent the linear regression fit of the kinetic data.

**Figure 6:**
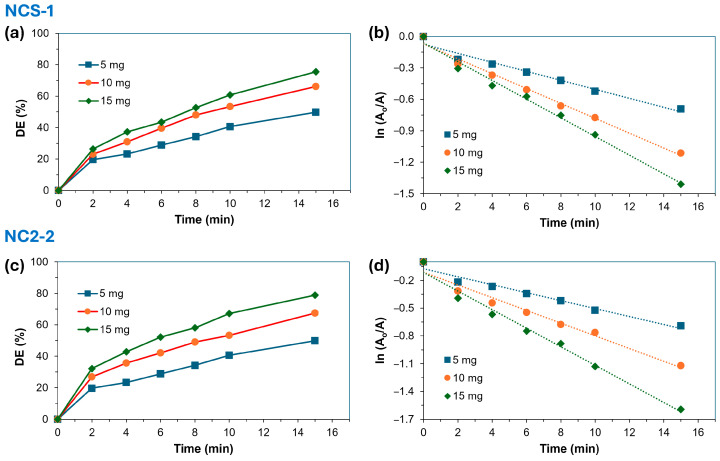
(**a**,**c**) Influence of the amount of NCS-1 and NCS-2 catalysts on the degradation efficiency (%) of MB and the diagrams of the (**b**,**d**) pseudo-first order degradation of MB using different amounts of NCS-1 and NCS-2 catalysts under light. The dotted lines in the kinetic diagrams represent the linear regression fit of the kinetic data.

Table 2 and Figure 5 and Figure 6 clearly demonstrate that the degradation efficiency (DE) of MB significantly increased with higher amounts of the NCS-1 and NCS-2 catalysts. However, the effectiveness of the catalysts was influenced by the prevailing light conditions. When NCS-1 and NCS-2 were exposed to both light and UV, they exhibited a similar trend under these conditions, yet their degradation efficiencies differed markedly. Under light, the degradation efficiencies for NCS-1 and NCS-2 were approximately 74.6% and 79.7%, respectively, while under UV illumination, these figures rose to 92.2% for NCS-1 and 92.9% for NCS-2. This suggests that UV illumination provides the most effective environment for degradation. When MB is exposed to 254 nm UV light, it primarily undergoes photodegradation through direct photolysis, resulting in the generation of highly reactive •OH radicals from the photolysis of water molecules in the solution.

These •OH radicals serve as potent oxidizing agents that decompose organic compounds like MB into smaller, less harmful molecules. In contrast, exposure to light leads to photodegradation through a combination of direct photolysis and indirect mechanisms such as photosensitization and reactions with oxygen and other environmental substances. Although direct photolysis under light can still produce •OH radicals, their formation efficiency may be lower due to the broader spectrum of light compared to UV light at 254 nm. Additionally, light can generate other reactive species like singlet oxygen (^1^O_2_) and superoxide radicals (O_2_^●−^), which may compete with •OH radicals in reacting with MB. These findings align with those reported by other researchers [36,37]. Regardless of the lighting conditions, the NCS-2 catalyst consistently demonstrated a higher efficiency than NCS-1.

### 2.3. Detection of Reactive Oxidative Species of NCS-2

**Figure 7:**
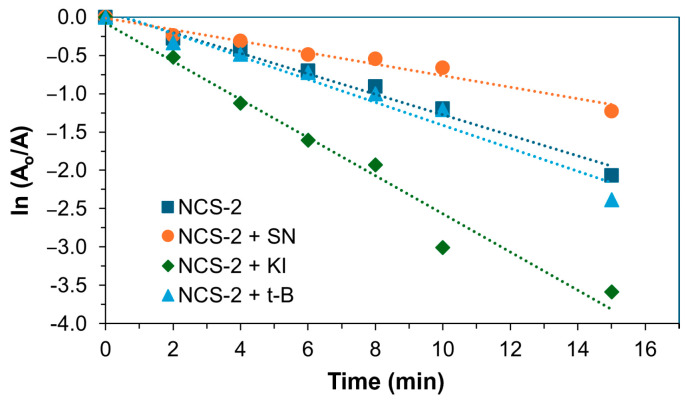
Influence of different scavengers on the pseudo-first order photocatalytic degradation efficiency of MB (H_2_O_2_-assisted) under UV light (254 nm) using 15 mg of NCS-2. The dotted lines in the kinetic diagrams represent the linear regression fit of the kinetic data.

**Figure 8:**
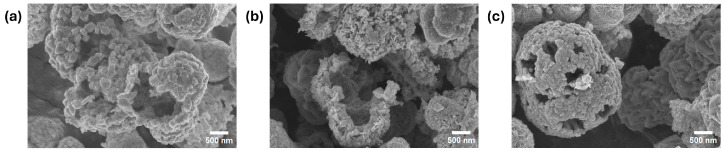
SEM images of NCS-2 after exposure to different scavengers: (**a**) SN, (**b**) KI, and (**c**) t-B scavengers.

**Figure 9:**
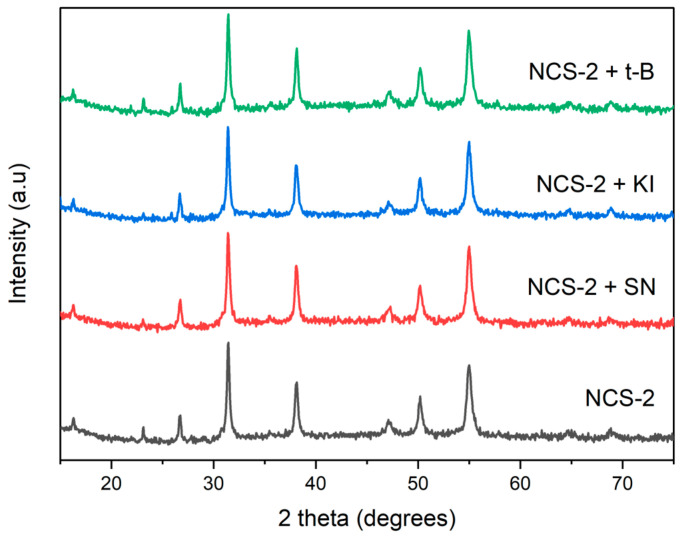
XRD pattern of NCS-2 after exposure to different scavengers.

In this study, a trapping experiment was conducted to assess the impact of scavenging agents on the reactive species responsible for the degradation of methylene blue (MB). The NCS-2 sample was chosen for investigation due to its superior efficiency compared to NCS-1. The photocatalytic process was performed under UV light irradiation, as this condition was most effective for MB degradation. Three scavenging agents were examined: potassium iodide (KI) as a scavenger for photogenerated holes (h^+^), silver nitrate (SN) as a scavenger for photogenerated electrons (e^−^), and tert-butanol (t-B) as a hydroxyl radical scavenger (•OH). During the experiment, 1 mL of 1 mM solutions of each scavenging agent was added to analyze their effects. Figure 7 illustrates the results of the scavenging test, demonstrating that the presence of an electron scavenger significantly inhibited the photodegradation rate of methylene blue. Silver nitrate, which captures the electrons generated during the photodegradation process, reduces the number of electrons available for MB degradation, resulting in the reactive species being predominantly responsible for the photodegradation process of MB. Interestingly, when MB was exposed to h^+^ and •OH scavengers, the rate of photodegradation increased. Typically, hydroxyl radicals play a crucial role in methylene blue degradation. However, the observed increase in the rate of degradation suggests that t-butanol might be interacting with other reactive species or mechanisms that enhance the degradation process. Similarly, potassium iodide captures the positive holes generated during photodegradation, which can lead to the formation of iodine radicals. These radicals may further contribute to the degradation of methylene blue, thereby increasing the overall degradation rate. Examining the surface morphology of the impact of scavengers on the NCS-2 catalyst, as illustrated in Figure 8, revealed some alterations or damage to the cluster formations. However, these structural changes did not significantly affect the XRD spectra, as shown in Figure 9. The peaks still corresponded to the cubic crystallographic structure of NiCo_2_S_4_, as indicated by JCPDS card no. 020-0782 [38].

### 2.4. Recyclability Experiments for NCS-2

A recyclability test was conducted to assess the practical application of the photocatalyst. The NCS-2 sample was recovered from the reaction mixture after the degradation process under UV light. After the initial cycle of degrading the MB dye, the catalyst was separated via centrifugation at 30,000 rpm for 10 min. The residue was washed twice with distilled water, then with 0.01 M HCl to remove any excess MB dye, and finally rinsed three more times with distilled water. The recycled catalyst was then dried in vacuo at 60 °C for 12 h.

**Figure 10:**
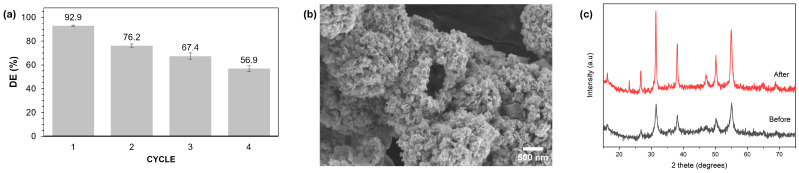
(**a**) Recyclability investigation of NCS-2 for the degradation of MB dye. (**b**) SEM image of NCS-2 after photocatalytic degradation. (**c**) XRD pattern before and after photocatalytic degradation.

Figure 10a illustrates that the photocatalytic performance of the catalyst significantly decreased with multiple reuse cycles. It achieved a performance of 92.9% in the first cycle, but this dropped to 56.9% by the fourth cycle. This reduction in efficiency may be due to sample loss during recovery and/or degradation of the catalyst, as evidenced by changes in its morphology. Figure 10b shows the appearance of holes in the catalyst, indicating physical changes. Additionally, the XRD pattern in Figure 10c confirmed that while all peaks corresponding to NiCo_2_S_4_ were still detectable, there was a new peak at 23.1°, suggesting that the catalyst had undergone slight changes.

### 2.5. Optimization of NCS-2 with Zeolite

The practical use of this photocatalyst is heavily influenced by its reusability. Our findings revealed that while NCS-2 performed impressively in a single cycle, its effectiveness diminished significantly after multiple uses. To enhance its reusability, the study investigated incorporating zeolite as a matrix for the catalyst, leveraging its cost-effectiveness and established use in wastewater treatment. Various samples were prepared with zeolite amounts ranging from 10 to 50 mg to identify the optimal quantity for creating a composite with both high and stable efficiency. As shown in Table 3, the degradation efficiency of the optimized NCS-2 catalyst combined with zeolite—referred to as NCS-2@Z—was highest with 20 mg of zeolite. This configuration achieved the greatest degradation efficiency and reaction rate. In contrast, pure zeolite alone yielded only a 58.3% degradation efficiency. Consequently, the NCS-2@Z formulation was chosen for further investigations into reusability and scavenging.

### 2.6. Characterization of the Optimized NCS-2@Z Catalyst

**Figure 11:**
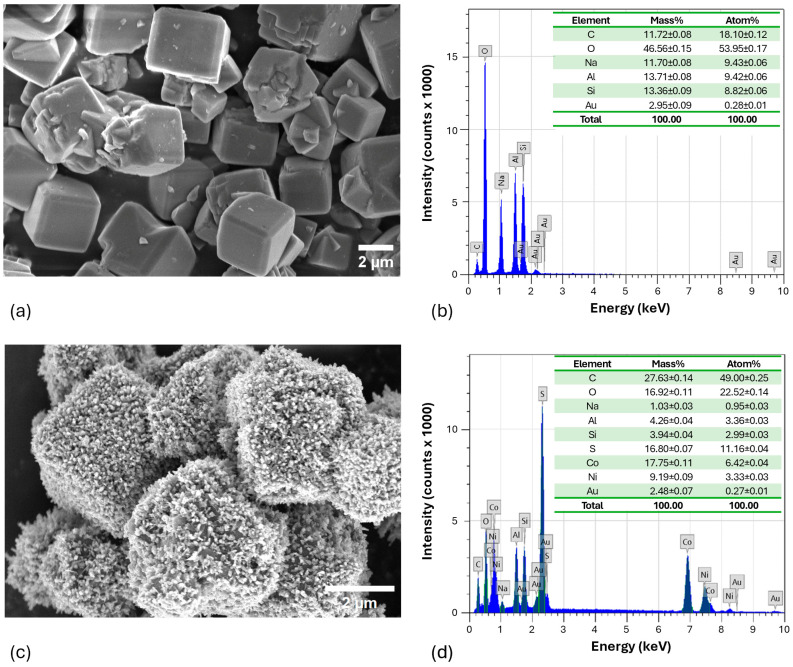
SEM images and EDX spectra with the elemental composition of (**a**,**b**) pure zeolite and (**c**,**d**) NCS-2@Z.

The morphological surface properties of pure zeolite and the NCS-2@Z composite were carefully examined by scanning electron microscopy, as shown in Figure 11. In Figure 11a, the pure zeolite shows a distinct cube-shaped structure with a size of about 1 to 5 µm. In contrast, Figure 11c illustrates the heterogeneous distribution of agglomerated NCS-2 on the surface of the zeolite matrix. This distribution of NCS changed the surface roughness of the zeolite, thereby increasing its surface area and active sites. These modifications are very beneficial for promoting efficient dye degradation [26]. To determine the elemental composition of the synthesized materials, a thorough analysis was conducted using electron dispersive spectroscopy, as illustrated in Figure 11b,d for pure zeolite and the NCS-2@Z composite, respectively. The analysis showed that the synthesized material contained only the targeted elements—Ni, Co, and S—corresponding to the NCS compound. Additional elements such as Na, Al, and Si were present due to the zeolite matrix. This indicates that the synthesized materials have a complex composition, incorporating both the desired NCS compound and components from the zeolite substrate.

**Figure 12:**
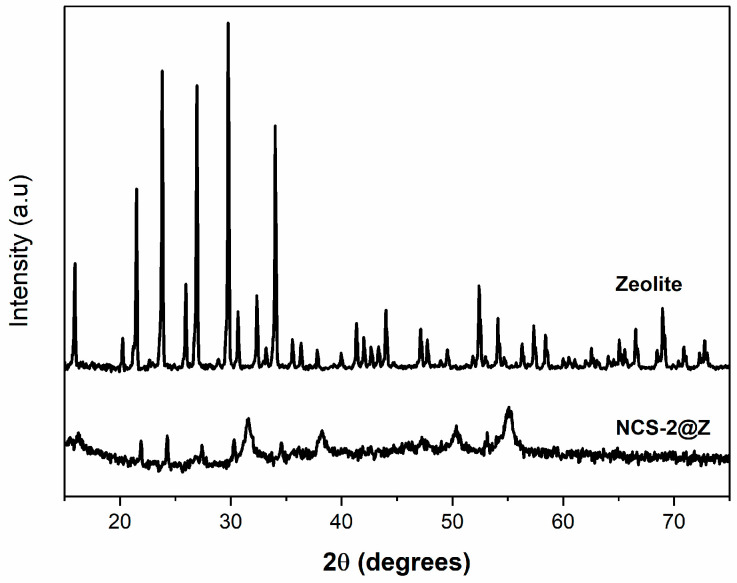
XRD spectra of pure zeolite and NCS-2@Z.

Furthermore, the crystallographic structures of pure zeolite and NCS-2@Z were evaluated by examining the diffraction peaks in the XRD spectra, as depicted in Figure 12. Here, peaks at 2θ values of 31.5°, 38.2°, 50.2°, and 55.2° can be seen, corresponding to the crystalline planes (311), (400), (511), and (440), respectively, from a cubic NiCo_2_S_4_ (JCPDS card no. 020-0782) [38]. Additionally, peaks at 15.9°, 21.5°, 23.8°, 27.4°, and 30.7° were attributed to the crystalline planes (311), (440), (537), (642), and (660), respectively, of zeolite [39]. These peaks indicate the formation of NiCo_2_S_4_ on the zeolite composites.

### 2.7. Recyclability Experiments for Optimized NCS-2@Z

**Figure 13:**
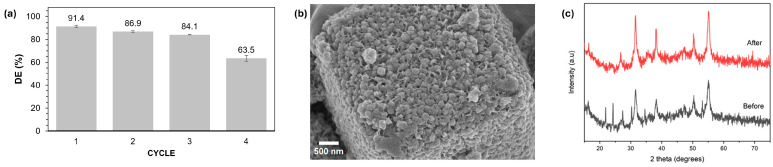
(**a**) Recyclability investigation of NCS-2@Z for the degradation of MB dye. (**b**) SEM image of NCS-2@Z after photocatalytic degradation. (**c**) XRD pattern before and after photocatalytic degradation.

To assess the stability and reusability of NCS-2@Z, a recyclability test was performed using the same procedure as for NCS-2. Figure 13a illustrates that the catalyst’s photocatalytic performance experienced a slight decline after being reused three times. There was a slight decrease from 91.4% to 84.1% after three cycles, whereas after the second cycle, the performance of the pure NCS-2 already decreased to 76.2%. This shows that the incorporation of zeolite into the NCS-2 framework significantly enhanced the catalyst’s stability and reusability. The presence of zeolite appears to provide additional structural support and prevent the leaching or degradation of active sites, thus maintaining a higher level of photocatalytic efficiency across multiple cycles. This improved performance stability indicates that NCS-2@Z is more robust compared to the pure NCS-2, making it a more reliable choice for applications requiring repeated use. This enhancement in stability is likely due to the improved dispersion of NCS within the zeolite matrix, as shown in Figure 13b. To investigate if there were any changes in the crystallographic structure of the matrix, the XRD pattern was analyzed, as depicted in Figure 13c. The analysis showed that certain peaks were absent after the photocatalytic process, particularly the peaks at 537 and 642, which are characteristic of the zeolite matrix. This absence suggests that there had been a structural change in the catalyst, potentially caused by the NCS covering the entire surface of the zeolite. These changes in the diffraction peaks correspond with the observed modifications in the material’s surface morphology, indicating that the surface structure of the zeolite had been significantly altered by the process.

## 3. Methodology

### 3.1. Materials

All chemicals and materials used in this study were of analytical grade and were not further purified. These included cobalt (II) nitrate hexahydrate (99%, AppliChem GmbH, Darmstadt, Germany), nickel (II) nitrate hexahydrate (97%, Sigma Aldrich, St. Louis, MO, USA), thiourea (VWR International, Leuven, Belgium), zeolite (Sigma Aldrich, St. Louis, MO, USA), methylene blue (MB dye, Sigma Aldrich, St. Louis, MO, USA), and ethanol (Sigma Aldrich, St. Louis, MO, USA).

### 3.2. Synthesis of NCS and Its Composites

For the synthesis of NCS, a mixture of Ni(NO_3_)_2_·6H_2_O, Co(NO_3_)_2_·6H_2_O, and thiourea with a Ni/Co/S ratio of 2:1:8 (in mmol) was dissolved in 15 mL of ethanol by sonication for 30 min. The solution was then transferred to a Teflon-lined stainless-steel autoclave and heated in a muffle furnace at 180 °C for 8 h. The mixture was then transferred to a tube and centrifuged at 8000 rpm for 10 min. The product was then collected, washed, filtered, and centrifuged with deionized water and ethanol. Finally, the solid product was dried in a vacuum at 60 °C for 12 h; it is referred to in this paper as NCS-1. To evaluate the effect of varying the molar ratio of Ni/Co on the photocatalytic performance for degrading methylene blue (MB), Ni/Co with a molar ratio of 1:2:8 (in mmol) was prepared, which was designated as NCS-2. Additionally, composites with zeolite were synthesized to investigate its influence on the stability and reusability of the photocatalyst. For the synthesis of NCS@Z, the same method was used for NCS but different amounts of zeolite (10, 20, 30, 40, and 50 mg) were incorporated into the solution, which was then sonicated for 30 min. The sample with Ni/Co/S at ratios of 2:1:8 (in mmol) and 1:2:8 (in mmol) with zeolite were designated as NCS-1@Z and NCS-2@Z, respectively.

### 3.3. Characterizations

Energy dispersive X-ray spectroscopy (EDX) was employed to analyze the composition of the materials, while scanning electron microscopy (SEM) was utilized to examine the particle size and morphology of the resulting materials. A JEOL JSM-IT200 (Tokyo, Japan) and ZEISS Crossbeam 540 (Jena, Germany) were utilized for the EDX and SEM analyses, respectively. Before conducting the analysis, the powdered samples underwent gold sputtering, employing a 20.0 mA sputter current, to a thickness of 5.0 nm and a tooling factor of 5.0. The powder X-ray diffraction (XRD) pattern analysis was performed using a Rigaku SmartLab system (Cedar Park, TX, USA) at 40 kV and 50 mA, ranging from 10 to 80° with a step size of 0.05°, and an X-ray source of Cu Kα at room temperature. The photocatalytic experiments were conducted utilizing an ASB-XE-175 fiberoptic light source with a xenon illuminator (Toption, Xi’an, China). For the UV irradiation experiments, a 254 nm wavelength (UVP, 3UV-38, 8 W) was employed. The optical absorption spectrum was recorded using an Avantes spectrometer (AvaSpec-ULS2048CL-EVO, Avantes B.V., Apeldoorn, The Netherlands) with a 25 μm slit size equipped with deuterium–halogen light source with a TTL shutter (AVALIGHT-DHC, Avantes B.V., Apeldoorn, The Netherlands) for the evaluation and monitoring of dye degradation.

### 3.4. Photocatalytic Experiments

In this study on the photocatalytic degradation of MB dye, three different conditions were used to evaluate the effectiveness of the photocatalyst in H_2_O_2_-assisted degradation: dark, UV exposure (254 nm), and simulation of artificial sunlight. To simplify the terminology in this article, these conditions will be referred to as dark, UV, and light, respectively.

For H_2_O_2_-assisted oxidation/degradation, a certain amount (5, 10, and 15 mg) of the synthesized catalyst was weighed and added to 25 mL of a 5 ppm MB dye solution (pH = 7.32) while stirring at 600 rpm under the various conditions considered in this study. After the addition of the catalyst, 1 mL of 30% H_2_O_2_ was added, and the mixture was stirred continuously for 15 min. At 2-, 4-, 6-, 8-, 10- and 15 min intervals, 3 mL of the samples was withdrawn. Each sample was then centrifuged at 3000 rpm for 1 min and the absorbance spectra of the supernatant were collected and measured using an Avantes UV–Vis spectrophotometer. The degradation percentage (*DE*%) of the MB dye by the catalyst was calculated using Equation (1).

(3) $$DE\%=\left(\frac{A_{o}-A_{t}}{A_{o}}\right)100$$

where *A_o_* and *A_t_* are the area under the curve in the 400–800 nm region initially and at time *t*, respectively. The area in the calculation was utilized instead of the common absorbance at a specific wavelength to account for all the resonance forms present in the system.

## 4. Conclusions

In this study, the successful synthesis of NCS using various ratios, both alone and in combination with zeolite, was demonstrated. The synthesized materials were characterized using techniques such as EDX analysis, SEM, and molar ratio determination. The EDX analysis confirmed the absence of impurities, while SEM revealed distinct morphological differences between pure zeolite, NCS, and the composite. Incorporating NCS significantly altered the surface roughness and active sites of the zeolite, enhancing the efficiency of dye degradation and improving the catalyst’s reusability. The degradation of methylene blue by the synthesized catalysts was evaluated under different light conditions. The pseudo-first order kinetics indicated faster reaction rates, particularly under UV radiation and visible light, suggesting that these light sources with specific wavelengths or energy levels promote the degradation process. Additionally, a positive correlation was observed between the amount of catalyst and the degradation rate. The highest degradation efficiency, along with increased stability and reusability, was achieved with NCS-2@Z, reaching 91.4% under UV irradiation. The study also identified e^−^ species as the primary agents responsible for MB degradation. These findings suggest that NCS@Z composites have significant potential as efficient catalysts for dye degradation, particularly under UV irradiation. Future research could explore their application to the degradation of other pollutants and extend their use in wastewater treatment and environmental protection.

## Figures and Tables

**Table 1:** Rate parameters for the degradation of MB by NCS-1 and NCS-2 catalysts under different light conditions.

Light Condition	Catalyst	Pseudo-1st Order		Pseudo-2nd Order	
		*r* ^2^	*k*_1_ (min^−1^)	*r* ^2^	*k*_2_ (min^−1^)
Dark	NCS-1	0.944	0.108	0.925	−0.041
	NCS-2	0.977	0.078	0.911	−0.027
UV	NCS-1	0.934	0.171	0.899	−0.078
	NCS-2	0.984	0.174	0.743	−0.065
Light	NCS-1	0.990	0.089	0.896	−0.028
	NCS-2	0.984	0.100	0.885	−0.033

**Table 2:** Rate and degradation parameters for the photooxidation of MB using different amounts of the catalysts under different light conditions.

Light Condition	Catalyst	Mass of Catalyst (mg)	DE%	Pseudo-1st Order	
				*r* ^2^	*k*_1_ (min^−1^)
UV	NCS-1	5	85.4	0.975	0.125
		10	85.6	0.998	0.134
		15	91.7	0.934	0.171
	NCS-2	5	74.9	0.985	0.086
		10	82.4	0.973	0.113
		15	93.4	0.984	0.174
Light	NCS-1	5	49.8	0.965	0.043
		10	66.2	0.989	0.071
		15	75.6	0.990	0.089
	NCS-2	5	49.8	0.965	0.043
		10	67.4	0.970	0.069
		15	78.8	0.984	0.100

**Table 3:** Rate and degradation parameters for the photooxidation of MB using different amounts of zeolite in a composite with the catalyst (NCS-2@Z) under UV irradiation.

Mass of Zeolite (mg)	DE%	Pseudo-1st Order	
		*r* ^2^	*k*_1_ (min^−1^)
10	86.0	0.993	0.132
20	91.4	0.980	0.155
30	88.5	0.988	0.136
40	87.5	0.992	0.135
50	85.9	0.989	0.129
pure zeolite	58.3	0.963	0.054

## Data Availability

The raw data supporting the conclusions of this article will be made available by the authors on request.
